# Supplementary material for: Plasma membrane LAT activation precedes vesicular recruitment defining two phases of early T-cell activation
Source: Nat Commun. 2018 May 22;9:2013. doi: 10.1038/s41467-018-04419-x (PMC5964120; doi:10.1038/s41467-018-04419-x)
Supplement: Supplementary file 1 — Supplementary Information [file 41467_2018_4419_MOESM1_ESM.pdf]

Plasma membrane LAT activation precedes vesicular recruitment defining two phases of early T cell activation

Lakshmi Balagopalan[1,*], Jason Yi[1], Tiffany Nguyen[1], Katherine M. McIntire[1], Adam S. Harned[2], Kedar Narayan[2] and Lawrence E. Samelson[1,*]

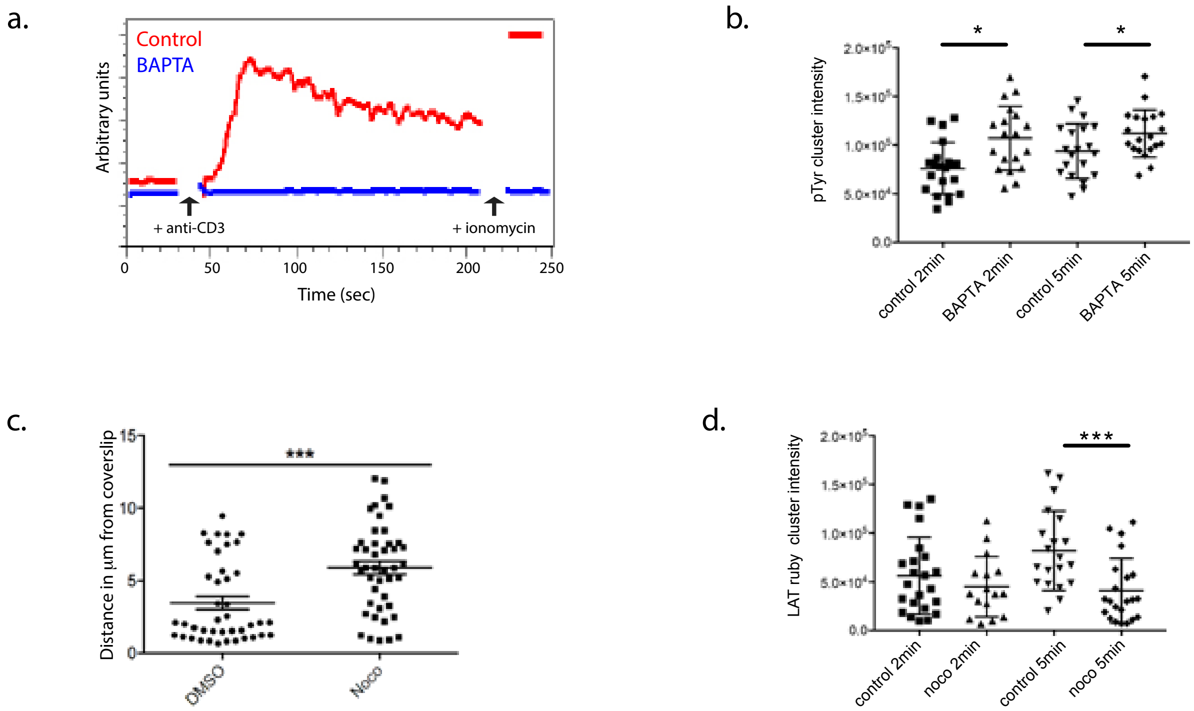

Supplementary Figure 1. BAPTA and nocodazole treatment of Jurkat cells. Related to Figure 1. **a.** Calcium flux of Indo-1AM loaded Jurkat cells treated with DMSO (control) or BAPTA in buffer containing EGTA (BAPTA). Cells were stimulated with anti-CD3 and ionomycin as indicated. **b.** Cluster intensity of pTyr microclusters in DMSO and BAPTA treated cells shown in Figure 1a-c were quantified. **c.** Jurkat cells transfected with LAT-RFP and emerald-VAMP7 were treated with DMSO or nocodazole (noco) and dropped onto stimulatory coverslips in medium containing DMSO or nocodazole and fixed 2 minutes after dropping. The distance of VAMP7 vesicles from the coverslip was measured and graphed. **d.** Cluster intensity of LAT-ruby microclusters in DMSO and nocodazole treated cells shown in Figure 1d-f were quantified.

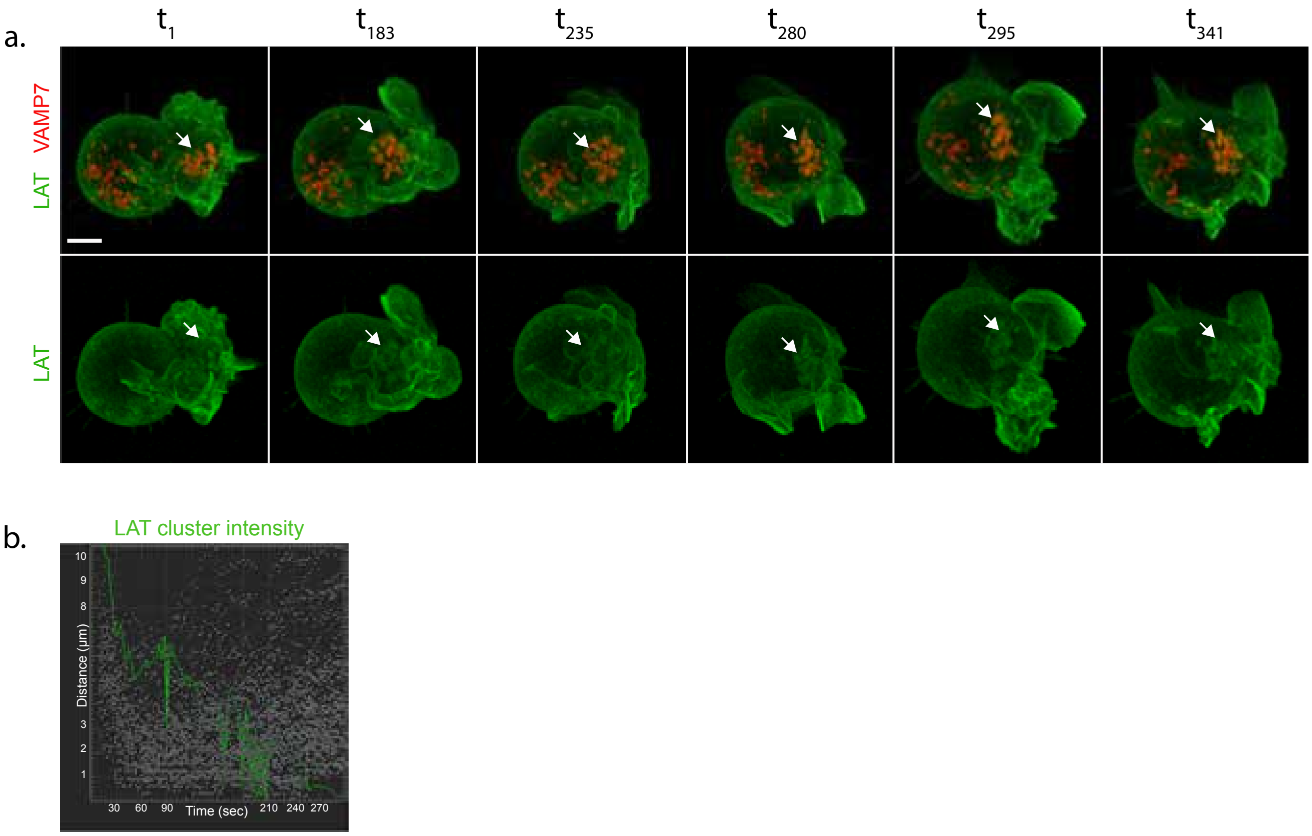

Supplementary Figure 2. Lattice Light Sheet image of unstimulated Jurkat cell and graph of LAT microcluster intensity relative to VAMP7 vesicle distance. Related to Figure 3.

a. Jurkat cells transfected with LAT-neon green and Halo-VAMP7 were dropped onto uncoated coverslips and imaged by Lattice Light Sheet Microscopy. t_0 indicates first time point of image collection. Top panel shows side view of cell. Arrow indicates VAMP7 vesicular pool that colocalizes with intracellular LAT. Scale bar equals 2µm. Bottom panel shows only LAT signal. **b.** Graph showing distances of individual VAMP7 vesicles from LAT clusters over time. It is the same graph as the one shown in Fig. 3c with vesicles in gray and intensity of the LAT microcluster surface overlaid in green.

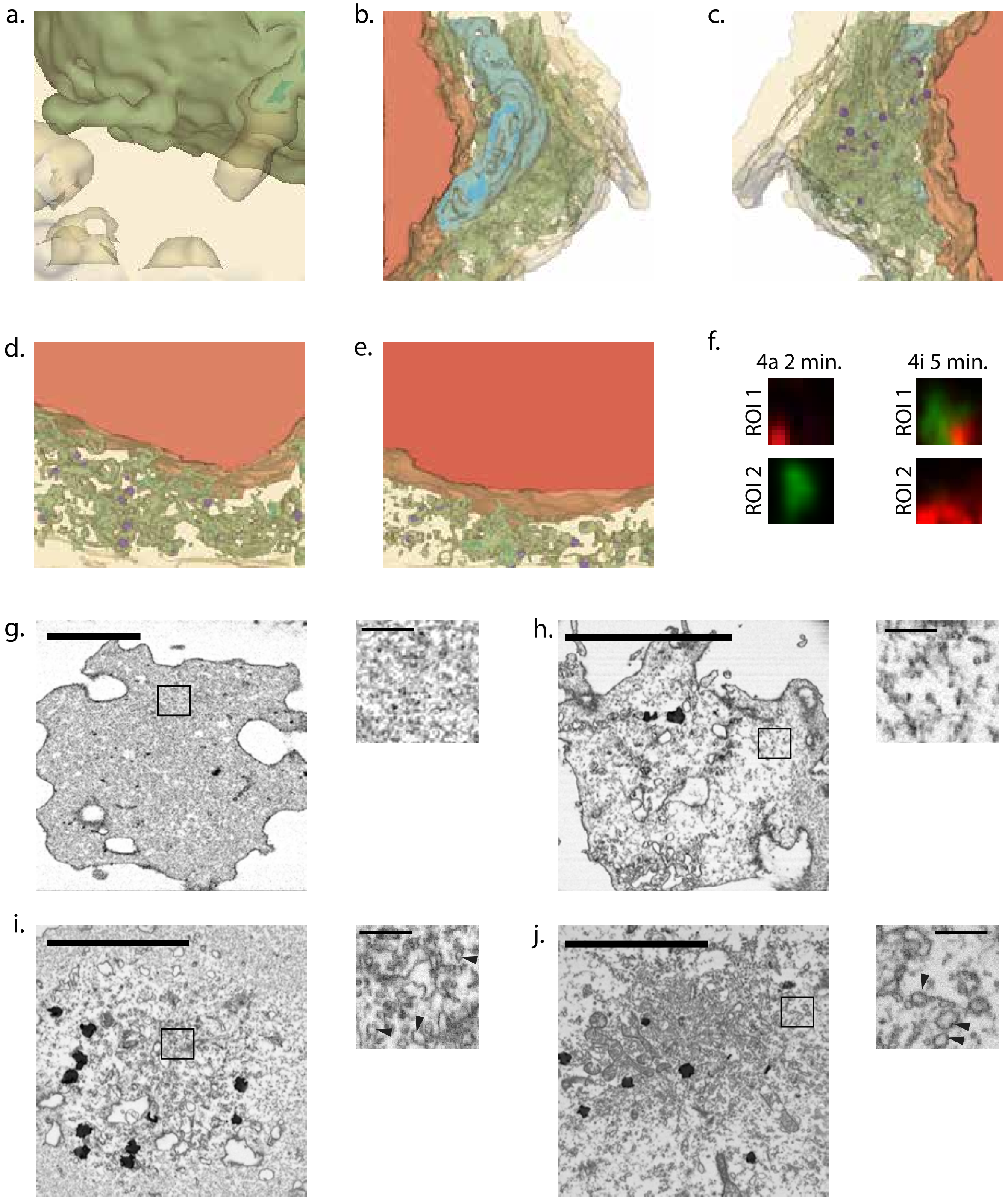

Supplementary Figure 3. FIB-SEM images of activated Jurkat cell. Related to Figure 4.
a-e. Segmented FIB-SEM subvolumes in which the nucleus and cytosol are shown in red and yellow respectively and membranes in these volumes are false colored green and vesicles are highlighted as purple spheres. **a** corresponds to Fig. 4d. **b** corresponds to Fig. 4g. A mitochondrion captured in this volume was segmented and colored blue. **c** is the same volume as b rotated to visualize the vesicles. **d** corresponds to Fig. 4k. **e** corresponds to Fig. 4m. **f.** Isolated red (pLAT) and green (VAMP-emerald) channels of the LM images corresponding to ROIs in Fig. 4a and i. **g-j.** *En face* FIBSEM images of Jurkat cells that have been dropped on to stimulatory coverslips for **(g and h)** 2 min and **(i and j)** 5 min. *En face* views of the entire cell (left panels) or zoomed in views of regions of interest (right panels) show an absence of vesicles at the synapse at 2 minutes and an abundance of vesicles (black arrowheads in zoomed-in panels) at the synapse at 5 minutes.

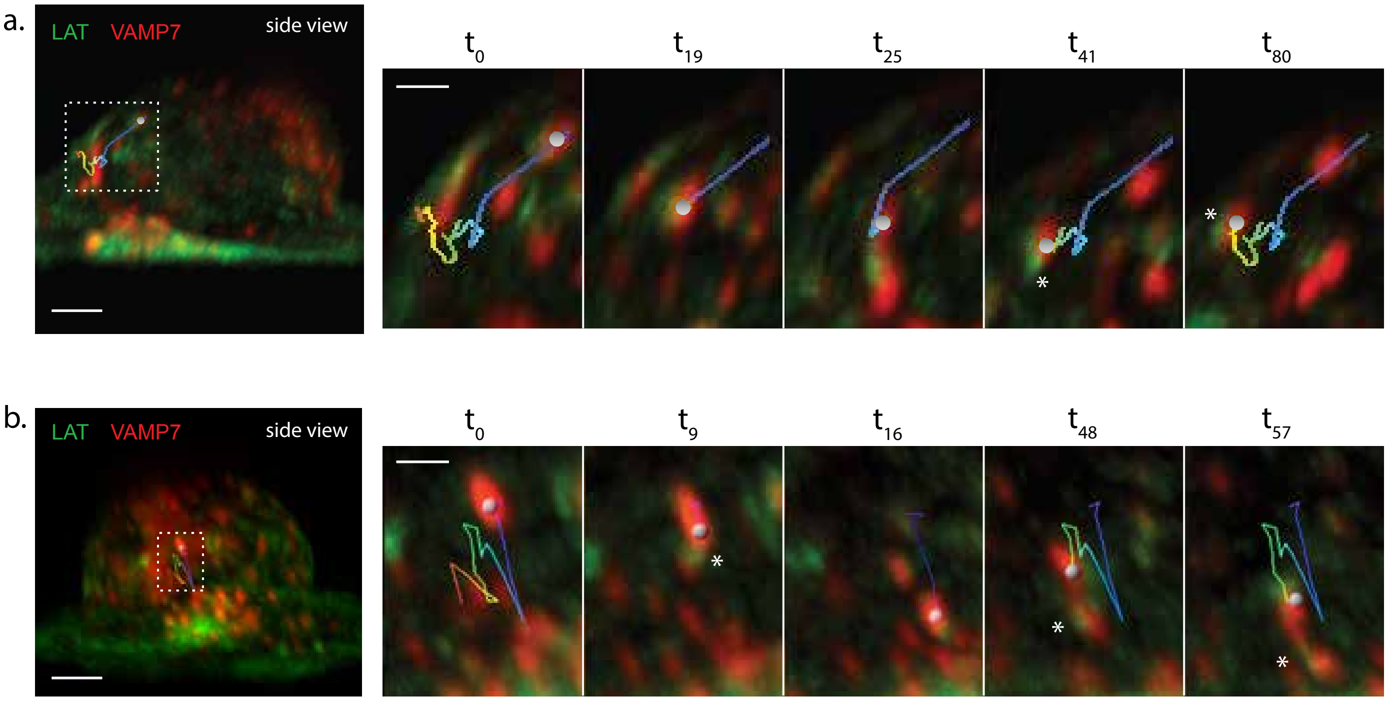

Supplementary Figure 4. Lattice Light Sheet image of Jurkat cell 5 minutes after activation. Related to Figure 6.
Lattice light sheet images of Jurkat cells transfected with LAT-neon green and Halo-VAMP7, dropped onto stimulatory coverslips and imaged 5 minutes after initial stimulation. **a.** A VAMP7 vesicle that tracks near the plasma membrane is shown with increases in LAT intensity at the junction of the VAMP7 vesicle and the plasma membrane highlighted by asterisks at t_{41} and t_{80}. **b.** Repeated fusion of VAMP7 vesicles with increases in LAT intensity at the junction of the vesicles highlighted by asterisks in $t_{9,}$ t_{48} and t_{57}. Left panels show a side view of the entire cell. The boxed region highlights a vesicle track. The tracked vesicle is a grey sphere. The entire track is shown and color-coded to indicate time, with the earliest time point in blue and the latest time point in red. Right panels show the zoomed-in view of the vesicle track. In the first panel at t_0, the entire track is shown. The remaining panels display 4 frames, each with a track that highlights just the previous 20 time-points. Scale bars for left panels equal 2µm and scale bars for right panels equal 0.5µm.

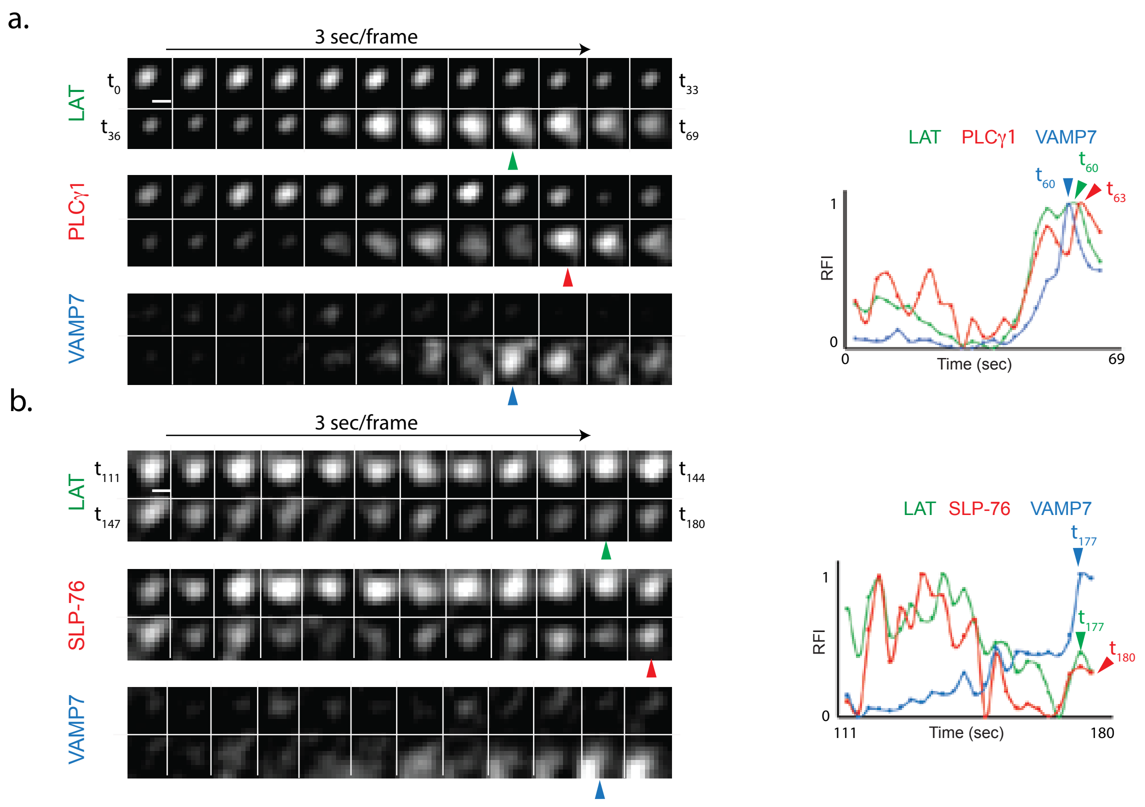

Supplementary Figure 5. Kinetic analysis of microcluster composition. Related to Figure 7. Jurkat cells were transfected with **(a)** LAT-ruby, PLCγ1-Halo and Emerald-VAMP7 or **(b)** LAT-ruby, SLP76-Halo and Emerald-VAMP7, dropped onto stimulatory coverslips and imaged by TIRF microscopy. Indicated time-points are shown with t_0 corresponding to the earliest observable time-point. Left panels show time-lapse montage at 3sec/frame of a single microcluster showing the fluorescence intensity kinetics of LAT, PLC γ1, SLP-76 and VAMP7. On the right are relative fluorescence intensity (RFI) plots corresponding to the timelapse montage on the left. Blue arrowheads indicate peak VAMP7 fluorescence, red arrowheads indicate peak PLC γ1 fluorescence **(a)** or increased SLP-76 fluorescence once VAMP7 is recruited **(b)** and green arrowheads indicate peak LAT fluorescence **(a)** or increased LAT fluorescence post VAMP7 recruitment **(b).** Scales bars equal 0.5μm.
