# Supplementary material for: Plasma membrane LAT activation precedes vesicular recruitment defining two phases of early T-cell activation
Source: Nat Commun. 2018 May 22;9:2013. doi: 10.1038/s41467-018-04419-x (PMC5964120; doi:10.1038/s41467-018-04419-x)
Supplement: Supplementary file 3 — Description of Additional Supplementary Files [file 41467_2018_4419_MOESM3_ESM.pdf]

Description of Additional Supplementary Files

Supplementary Movie 1. TIRF movie showing LAT recruitment to microclusters precedes VAMP7. Related to Figure 2a
TIRF movie of a T cell expressing LAT-RFP (red) and GFP-VAMP7 (green).

Supplementary Movie 2. TIRF movie showing Grb2 recruitment to microclusters precedes VAMP7. Related to Figure 2d
TIRF movie of a T cell expressing Grb2-Apple (green) and Halo-VAMP7 (red).

Supplementary Movie 3. Lattice Light Sheet Microscope movie showing LAT and VAMP7 distribution in an unstimulated cell. Related to Supplementary Figure 2a.
Time-lapse maximum intensity projections of lattice light-sheet images of a side view of a T cell expressing LAT-neongreen (green) and Halo-VAMP7 (red) as it migrates on a nonstimulatory coverslip.

Supplementary Movie 4. Lattice Light Sheet Microscope movie showing LAT and VAMP7 distribution in a cell dropped on a stimulatory coverslip. Related to Fig. 3a.
Time-lapse maximum intensity projections of lattice light-sheet images of a side view of a T cell expressing LAT-neongreen (green) and Halo-VAMP7 (red), dropped on a Hit3a (anti-CD3ε) coated stimulatory coverslip.

Supplementary Movie 5. Segmentation and distance analysis of Lattice Light Sheet Microscope movie showing LAT and VAMP7 distribution. Related to Fig. 3b.
Segmentation of data in Movie S4. Surfaces were generated of LAT clusters (green), VAMP7 vesicles (red), spots were generated within VAMP7 surfaces (cyan and purple) and distances of VAMP7 vesicles to LAT clusters were mapped over time (distance scale in Fig. 3b scale bar).

Supplementary Movie 6. Lattice Light Sheet Microscope movie showing a T cell expressing Grb2 and LAT interacting with an SEE pulsed Raji B cell. Related to Fig. 3.
Time-lapse maximum intensity projections of lattice light-sheet images of a side view of a T cell expressing LAT-Halo (green) and Grb2-GFP (red), interacting with an SEE pulsed Raji B cell (blue).

Supplementary Movie 7. Lattice Light Sheet Microscope movie showing a T cell expressing LAT and VAMP interacting with two SEE pulsed Raji B cells. Related to Fig. 3d.
Lattice Light Sheet Microscope movie of a T cell expressing LAT-neongreen (green) and Halo-VAMP7 (red), interacting with two SEE pulsed Raji B cell (blue).

Supplementary Movie 8. Focused Ion Beam Electron Microscopy (FIB-SEM) stack of ROI 1 shows no vesicles near microclusters at early times after stimulation. Related to Fig. 4c.
FIB-SEM stack of x-z slices of ROI 1 shown in Figure 4b corresponding to a phospho-LAT microcluster by LM. No vesicles are seen in the stack.

Supplementary Movie 9. Segmented volume of Focused Ion Beam Electron Microscopy (FIB-SEM) stack of ROI 1 shown in Supplementary Movie 8. Related to Fig. 4e.
Segmented volume of ROI 1 shown in Figure 4b corresponding to a phospho-LAT microcluster by LM. No vesicles are seen in the volume.

Supplementary Movie 10. Focused Ion Beam Electron Microscopy (FIB-SEM) stack of ROI 2 showing vesicles. Related to Fig. 4f.
FIB-SEM stack of x-z slices of ROI 2 shown in Figure 4b corresponding to emerald-VAMP7 signal by LM. Several small vesicles are seen in the volume.

Supplementary Movie 11. Segmented volume of Focused Ion Beam Electron Microscopy (FIB-SEM) stack of ROI 2 shown in Supplementary Movie 10. Related to Fig. 4h.
Segmented volume of x-z slices of ROI 2 shown in Figure 4b corresponding to a phospho-LAT microcluster by LM. Several small vesicles are seen in the volume.

Supplementary Movie 12. Focused Ion Beam Electron Microscopy (FIB-SEM) stack of ROI 1 showing vesicles near the synapse at late times (5 min) after stimulation. Related to Fig. 4k.
FIB-SEM stack of x-z slices of ROI 1 shown in Figure 4j. Several small vesicles located close to the synaptic surface are seen in the volume.

Supplementary Movie 13. Focused Ion Beam Electron Microscopy (FIB-SEM) stack of ROI 2 showing vesicles near the synapse at late times (5 min) after stimulation. Related to Fig. 4m.
FIB-SEM stack of x-z slices of ROI 2 shown in Figure 4j. Several small vesicles located close to the synaptic surface are seen in the volume.

Supplementary Movie 14. Segmented volume of Focused Ion Beam Electron Microscopy (FIB-SEM) stack of ROI 1 shown in Supplementary Movie 12. Related to Fig. 4l.
Segmented volume of x-z slices of ROI 1 shown in Figure 4j corresponding to a phospho-LAT microcluster by LM. Several small vesicles located close to the synaptic surface are seen in the volume.

Supplementary Movie 15. Segmented volume of Focused Ion Beam Electron Microscopy (FIB-SEM) stack of ROI 2 shown in Supplementary Movie 13. Related to Fig. 4n.
Segmented volume of x-z slices of ROI 2 shown in Figure 4j corresponding to a phospho-LAT microcluster by LM. Several small vesicles located close to the synaptic surface are seen in the volume.

Supplementary Movie 16. TIRF-SIM movie showing ZAP-70 and VAMP7 distribution in a cell dropped on a stimulatory coverslip. Related to Fig. 5a.
TIRF-SIM movie of a T cell expressing ZAP-Apple (green) and Halo-VAMP7 (red), dropped on a stimulatory coverslip and imaged 5 minutes after activation.

Supplementary Movie 17. Zoomed-in TIRF-SIM movie showing track of VAMP7 vesicle on ZAP-70 microcluster. Related to Fig. 5b.

Zoomed-in area of Supplementary Movie 16 showing a VAMP7 vesicle (red - indicated as a grey sphere) tracking on a ZAP70 microcluster (green). The track is color-coded to indicate time with the earliest time point in blue and the latest time point in red.

Supplementary Movie 18. Zoomed-in TIRF-SIM movie showing a VAMP7 vesicle moving between ZAP-70 microclusters. Related to Fig. 5c.
Zoomed-in area of Supplementary Movie 16 showing a VAMP7 vesicle (red - indicated as a grey sphere) tracking between multiple ZAP70 microclusters (green). The track is color-coded to indicate time with the earliest time point in blue and the latest time point in red.

Supplementary Movie 19. Zoomed-in region of fast capture TIRF-SIM movie showing a VAMP7 vesicle moving between ZAP-70 microclusters. Related to Fig. 5.
Zoomed-in area of a TIRF-SIM movie in which ZAP70 microclusters (green) were collected at the beginning and end of the movie and VAMP7 (red) vesicle dynamics were captured every 200msec and overlaid over the ZAP70 microclusters.

Supplementary Movie 20. Tracks of segmented VAMP7 vesicles moving between segmented ZAP-70 microclusters. Related to Fig. 5d.
Zoomed-in area of a TIRF-SIM movie showing tracks (yellow) of two VAMP7 vesicles (red segments) moving between ZAP70 microclusters (green segments). Tracks 145 and 213 are shown.

Supplementary Movie 21. TIRF-SIM movie of an entire cell showing VAMP7 vesicles moving on microtubules. Related to Fig. 5g.
TIRF-SIM movie of a T cell expressing Grb2-Apple (green), Halo-VAMP7 (red) and EMTB-GFP (blue) dropped on a stimulatory coverslip and imaged 5 minutes after activation.

Supplementary Movie 22. Zoomed-in TIRF-SIM movie showing a VAMP7 vesicle moving on a microtubule. Related to Fig. 5h.
A zoomed-in region of TIRF-SIM movie showing a VAMP7 vesicle (red) showing a curvilinear track on a microtubule (blue).

Supplementary Movie 23. Zoomed-in TIRF-SIM movie showing a VAMP7 vesicle moving on a microtubule between two Grb2 microclusters. Related to Fig. 5i.
A zoomed-in region of TIRF-SIM movie showing a VAMP7 vesicle (red) moving on a microtubule (blue) between two Grb2 microclusters (green).

Supplementary Movie 24. Zoomed-in TIRF-SIM movie showing a nocodazole-treated cell. Related to Fig. 5.
TIRF-SIM movie of a nocodazole-treated T cell expressing Grb2-Apple (green), Halo-VAMP7 (red) and EMTB-GFP (blue) dropped on a stimulatory coverslip in medium containing nocodazole.

Supplementary Movie 25. Lattice Light Sheet Microscope movie showing a VAMP7 vesicle tracking near the plasma membrane. Related to Supplementary Figure 4a.
Time-lapse maximum intensity projections of lattice light-sheet images of a side view of a T cell expressing LAT-neongreen (green) and Halo-VAMP7 (red), dropped on a

stimulatory coverslip and imaged 5 minutes after activation. A VAMP7 vesicle tracking near the plasma membrane is shown.

Supplementary Movie 26. Lattice Light Sheet Microscope movie showing repeated "kissing" of VAMP7 vesicles. Related to Supplementary Figure 4b.
Time-lapse maximum intensity projections of lattice light-sheet images of a side view of a T cell expressing LAT-neongreen (green) and Halo-VAMP7 (red), dropped on a stimulatory coverslip and imaged 5 minutes after activation.

Supplementary Movie 27. Lattice Light Sheet Microscope movie tracking a vesicle moving towards the synapse and captures a LAT "flare" at the synapse. Related to Fig. 6a.
Time-lapse maximum intensity projections of lattice light-sheet images of a side view of a T cell expressing LAT-neongreen (green) and Halo-VAMP7 (red), dropped on a stimulatory coverslip and imaged 5 minutes after activation.

Supplementary Movie 28. Lattice Light Sheet Microscope movie tracking a vesicle moving from the center to the periphery of the synapse and captures a LAT "flare" at the synapse. Related to Fig. 6b.
Time-lapse maximum intensity projections of lattice light-sheet images of a side view of a T cell expressing LAT-neongreen (green) and Halo-VAMP7 (red), dropped on a stimulatory coverslip and imaged 5 minutes after activation.
